# Diagnostic Utility of Integrated ^11^C-Pittsburgh Compound B Positron Emission Tomography/Magnetic Resonance for Cerebral Amyloid Angiopathy: A Pilot Study

**DOI:** 10.3389/fnagi.2021.721780

**Published:** 2021-11-25

**Authors:** Yan Chang, Jiajin Liu, Liang Wang, Xin Li, Zhenjun Wang, Mu Lin, Wei Jin, Mingwei Zhu, Baixuan Xu

**Affiliations:** ^1^Department of Nuclear Medicine, The First Medical Centre, Chinese PLA General Hospital, Beijing, China; ^2^PET/CT, Jixi Ji Mine Hospital, Jixi, China; ^3^Department of Interventional Radiology, The First Medical Centre, Chinese PLA General Hospital, Beijing, China; ^4^Department of Radiology, The First Medical Centre, Chinese PLA General Hospital, Beijing, China; ^5^MR Collaboration, Diagnostic Imaging, Siemens Healthcare Ltd., Shanghai, China; ^6^Department of Pathology, The First Medical Centre, Chinese PLA General Hospital, Beijing, China; ^7^Department of Neurology Medicine, The Second Medical Centre, Chinese PLA General Hospital, Beijing, China

**Keywords:** cerebral microbleed, cerebral amyloid angiopathy, amyloid, positron emission tomography/magnetic resonance imaging, Alzheimer’s disease

## Abstract

**Objective:** We aimed to compare amyloid deposition at the lobar cerebral microbleed (CMB) sites of cerebral amyloid angiopathy (CAA), Alzheimer’s disease (AD), and cognitively normal healthy controls (NC) and to propose a novel diagnostic method for differentiating CAA patients from AD patients with integrated ^11^C-Pittsburgh compound B (PIB) positron emission tomography (PET)/magnetic resonance (MR) and assess its diagnostic value.

**Methods:** Nine CAA, 15 AD patients, and 15 NC subjects were enrolled in this study. Each subject underwent an ^11^C-PIB brain PET/MR examination. Susceptibility weighted imaging was assessed to detect CMB locations, and standardized uptake value ratios (SUVRs) were measured at these sites. Cortical PIB distributions were quantitatively evaluated. Patients with CAA, AD, and NC subjects were compared with global and regional cortical SUVRs at CMB cites. The diagnostic accuracy of MRI, PIB-PET, and PET/MR in differentiating CAA and AD was evaluated.

**Results:** Lobar CMBs were detected in all the CAA patients, eight of the 15 AD patients (53.3%), and four of the 15 NC subjects (26.7%), respectively. The PIB deposition at CMB sites was significantly higher in CAA patients compared with AD patients and NC subjects in terms of SUVR (1.72 ± 0.10 vs. 1.42 ± 0.16 and 1.17 ± 0.08; *p* < 0.0001). The PIB deposition was associated with CMB locations and was greatest in the occipital and temporal regions of CAA patients. The global cortical PIB deposition was significantly higher in CAA than in NC subjects (1.66 ± 0.06 vs. 1.21 ± 0.06; *p* < 0.0001) and significantly lower than in AD patients (1.66 ± 0.06 vs. 1.86 ± 0.17; *p* < 0.0001). In contrast, the occipital/global PIB uptake ratio was significantly increased in CAA (occipital/global ratio, 1.05 ± 0.02) relative to AD patients (1.05 ± 0.02 vs. 0.99 ± 0.04; *p* < 0.001). PET/MR had a higher accuracy (sensitivity, 88.9%; specificity, 93.3%) than separate PET and MR.

**Conclusion:** Our results indicate that the CMBs occur preferentially at loci with concentrated amyloid. By combining lobar CMBs with regional cortical amyloid deposition, the proposed workflow can further improve CAA diagnostic accuracy compared to each method alone. These findings improve our knowledge regarding the pathogenesis of CMBs and highlight the potential utility of PIB-PET/MR as a non-invasive tool for distinguishing CAA and AD patients.

## Introduction

Cerebral amyloid angiopathy (CAA) is an extremely common small vessel disease (SVD) of the brain in elderly individuals. It is caused by progressive amyloid-β protein accumulation within cerebral vessels, particularly involving the cortical and leptomeningeal vessel walls (Vinters, 1987; Viswanathan and Greenberg, 2011; Charidimou et al., 2012). CAA is an important common cause of spontaneous lobar intracerebral hemorrhage (ICH), having a high risk of morbidity and mortality (Charidimou et al., 2012). Currently, the Boston criteria are widely applied for the clinical diagnosis of CAA-related ICH, as they are easy to use and have been validated with histopathologic examinations (Linn et al., 2010). Patients with more than two strictly, lobar ICH/cerebral microbleeds (CMBs) without an identified cause were diagnosed as “probable CAA”; these represent late and irreversible brain injuries. Patients with only one ICH or CMB are interpreted as “possible CAA.” For these patients, a diagnosis of early-stage CAA would change the medical management and reduce the risk of new ICHs (Shoamanesh et al., 2017).

As a main hallmark of CAA, CMBs have been demonstrated to increase with age by the prior population-based studies, approaching 40% in those 80 years and older (Poels et al., 2010; Romero et al., 2014). Magnetic resonance imaging (MRI) is the primary neuroimaging modality for the detection of CMBs. Studies have shown that the various CMB distribution patterns reflect different vascular pathologies (Yakushiji, 2015). The presence of multiple, strictly lobar CMB distributions is positively related to advanced CAA (Smith and Greenberg, 2003; Van Etten, 2014), whereas, deep CMBs are thought to be associated with hypertensive damage of deep penetrating arteries (Greenberg et al., 2009). Meanwhile, strictly lobar CMBs are not related to classic vascular risk factors (Vernooij et al., 2008; Romero et al., 2014), which further reinforces possible associations with CAA. Martinez-Ramirez et al. (2015) investigated the diagnostic value of solely lobar CMBs, and the positive predictive values were 87.5 and 25% for hospital and general populations, respectively, which suggests that the CMBs should be associated with other supporting clinical evidence. Positron emission tomography (PET) with ^11^C-Pittsburgh compound B (PIB) imaging can detect cerebrovascular amyloid depositions and potentially enable CAA diagnoses even earlier than when MRI is used. However, due to the low resolution of PET, vascular amyloid cannot be differentiated from parenchymal amyloid. Consequently, ^11^C-PIB-PET has a low specificity for CAA due to frequent parenchymal amyloid depositions in healthy elderly people, reflecting incipient Alzheimer’s disease (AD) (Baron et al., 2014).

Cerebral amyloid angiopathy diagnosis might be improved by combining the complementary information of lobar CMBs and β amyloid burden. The newly emerging PET/MR imaging method integrates the advantages of PET and MR and has been used to diagnose and monitor patients with various neurodegenerative conditions (Mainta et al., 2017). To our knowledge, no study has investigated the diagnostic value of combining amyloid deposition and CMB distribution, specifically in CAA and AD patients and cognitively normal healthy controls (NC) using integrated PET/MR. In this pilot study, we aimed (1) to compare amyloid deposition at strictly lobar CMBs in patients with CAA, AD, and NC subjects and (2) to propose a novel diagnostic method to differentiate CAA patients from AD patients with PIB-PET/MR.

## Materials and Methods

### Study Participants

A total of 39 subjects (9 CAA, 15 AD patients, and 15 NC subjects) aged 55–86 years were prospectively enrolled in this study. We included all eligible patients who visited the Department of Neurologic Medicine in Chinese PLA General Hospital and who underwent brain PIB PET/MR examinations between 2017 and 2019. The clinical diagnosis of possible or probable CAA is currently based on the Boston criteria, which have been validated against the pathologic gold standard. Scrutiny of the brain MRI scans of all patients identified only eight fulfilled the Boston criteria for possible or probable CAA, and one patient had probable CAA with supportive pathology (Patient 6). The remaining patients were not confirmed with pathology due to the unavailability of the autopsy. Mini-Mental State Examination (MMSE) scores were administered to eight of the nine CAA patients. One patient was unable to provide meaningful MMSE examination due to severe hearing impairment. MMSE testing revealed seven CAA patients had no cognitive deficits, while one patient had an MMSE score of <17, suggesting a severe decline in cognitive function. Spontaneous ICHs were observed in seven of the nine CAA. The CAA patient demographics and clinical characteristics are summarized in Table 1. None of the NC subjects had cognition impairments or subjective memory complaints with MMSE scores of ≥28. Clinically probable AD patients (*n* = 15) met the National Institute of Neurological and Communicative Disorders and Stroke and the AD and Related Disorders Association (NINCDS–ADRDA) criteria (McKhann et al., 1984), and the MMSE scores ranged from 18 to 26.

**TABLE 1:** The demographics and clinical characteristics of patients with cerebral amyloid angiopathy (CAA).

Patient	Age (years)	M/F	I-ICH	Main clinical presentation	VRF	WMH (Fazekas Scale)	Lobar MBs	cSS	MMSE	Timing of PIB-PET (months)
1	80	M	R Front	Left sided paresthesia weakness, Amnesia	D, dyslipidaemia	3	1	Focal	27	Positive (24)
2	85	M	R Par	Dizzy, dysphasia, left sided paresthesia weakness	AF, AHT	3	3	Focal	29	Positive (12)
3	89	M	L Front	Dizzy	AHT, ex-smoker, dyslipidaemia	2	2	–	27	Positive (24)
4	80	F	L Occ	Amnesia	D, AF, AHT, dyslipidaemia	3	1	Focal	30	Positive (30)
5	92	M	L Par	Amnesia, abnormal behavior	Ex-smoker	3	5	Disseminated	N/A	Positive (18)
6	82	M	R Temp-Par	Left limb movement disorder	Ex-smoker	3	>5	Disseminated	17	Positive (20)
7	65	M	R Par	Dizzy	D, AHT, dyslipidaemia	2	>5	Focal	30	Positive (17)
8	74	M	–	Left arm paresthesia weakness	AHT	2	1	–	30	Positive (20)
9	69	F	–	Right arm and limb paresthesia weakness	AHT	2	1	–	30	Positive (22)

*CAA, cerebral amyloid angiopathy; PET, positron emission tomography; PIB, Pittsburgh compound B; M/F, gender; I-ICH, lobar intracerebral hemorrhage; VRF, vascular risk factors; WMH, white matter hyperintensity; CMBs, cerebral microbleeds; cSS, cortical superficial siderosis; MMSE, Mini-Mental State Examination; R, right; Front, frontal cortex; Occ, occipital cortex; D, diabetes; Disseminated, cSS affecting ≥ 4 sulci; L, left; Par, parietal cortex; AF, atrial fibrillation (paroxystic); AHT, arterial hypertension; Focal, cSS affecting < 4 sulci; Temp, temporal cortex.*

### Histology

Patient 6 received a right temporal and parietal intracerebral hemorrhage removal. Hematoma was evacuated and the specimens were embedded in paraffin, and stained using the Congo red method. The tissue sections were deparaffinized and washed in water, then submerged in a Congo red aqueous solution for 5–10 min, and then they were rinsed in water, rehydrated, and differentiated through saturated lithium carbonate solution. The sections were stained with hematoxylin for 2–3 min, and rinsed twice in water, dehydrated through serial alcohols, transparented with xylene, and mounted with resinous. The sections stained by Congo red were observed under polarized light.

### Integrated Positron Emission Tomography/Magnetic Resonance Imaging Acquisitions

All subjects underwent brain examinations using a whole-body hybrid PET/MR scanner (Biograph mMR, Siemens Healthcare, Erlangen, Germany) with a dedicated 16-channel head coil. The MRI scanning protocol included the following sequences: a 3D-T1-MPTAGE-sequence [sagittal orientation; repetition time (TR), 1800 ms; echo time (TE), 2.44 ms; slice thickness (ST), 1 mm; matrix size, 256 × 256], T2-weighted-fluid-attenuated inversion recovery (FLAIR) sequence (axial orientation; TR 8,000 ms; TE 94 ms; inversion time: 2,370 ms; ST 5 mm; matrix size 256 × 256), diffusion-weighted imaging (DWI) sequence (axial orientation; TR 5,100 ms; TE 137 ms; *b*-values = 0, 1,000 s/mm^2^; ST 4 mm; matrix size 164 × 170, averages 6), and susceptibility-weighted imaging (SWI) sequence (axial orientation; TR 26 ms; TE 20 ms; ST 1.2 mm; matrix size 280 × 320). For the head, PET was scanned using a one-bed position (axial field of view 25.8 cm) simultaneously. Three-dimensional (3D) mode acquisitions were obtained for 20 mins.

^11^C-PIB was intravenously injected at 4.44–5.55 MBq/kg. ^11^C-PIB-PET images were obtained 40–60 mins after the injections. The PET data were reconstructed using common Poisson-ordered subset expectation-maximization algorithms with three iterations Twenty-one subsets were obtained using a Gaussian filter of 4 mm full-width at half-maxima (FWHM) and 344 × 344 voxels.

### Positron Emission Tomography Data Analyses

To obtain standardized uptake value ratios (SUVRs) for the PIB-PET scans, voxel intensities were normalized with the mean intensity of the cerebellar cortices as a reference region. In order to unify the CMB-based SUVR measurements, a circular ROI with a fixed diameter of 10 mm was drawn at each CMB site, and the SUVR was calculated within the ROI. Global Aβ depositions were assessed from the volume-weighted mean SUVRs at the following cortical ROIs: precuneus, frontal, parietal, lateral temporal, occipital, and anterior and posterior cingulate. According to a previous study, SUVRs larger than 1.5 were defined as global PIB positive (Villemagne et al., 2011). The regional cortical PIB SUVRs were obtained for the occipital, frontal, and temporal regions. The occipital/global, frontal/global, and lateral temporal/global SUVR ratios were also calculated to demonstrate the regional distributions of PIB deposition. Receiver operating characteristic (ROC) curves were used to determine the optimal cutoff values for global SUVRs and occipital/global ratios for differentiating CAA from AD cohorts. The ROC curves were also used to assess the accuracy of differentiation between the cohorts.

### Definition and Assessment of Small Vessel Disease Markers on Magnetic Resonance Imaging

Two experienced readers, blinded to the diagnoses and all other clinical data, reviewed the MR images. A consensus was sought in cases with a discrepancy. All structural imaging SVD markers were rated according to the current consensus guidelines (Wardlaw et al., 2013). Lobar CMBs, defined as homogeneous and focal round lesions with signal losses on the SWI images, were different from vascular flow voids, calcifications, cavernous malformations, and basal ganglia mineralizations, and located exclusively in lobar areas. cSS was defined as a homogenous curvilinear signal loss on SWI, outlining the superficial layers of the cerebral cortex (Charidimou et al., 2017). The distribution and severity of cSS were classified as absent, focal (restricted to ≤ 3 sulci), and disseminated (≥4 sulci) (Linn et al., 2010). Enlarged perivascular spaces (PVSs) were assessed in line with the Standards for Reporting and Imaging of Small Vessel Disease (STRIVE) (Wardlaw et al., 2013) and rated on axial T2-weighted MRI images in the basal ganglia (BG) and centrum semi vale (CSO), using a validated 4-point visual rating scale (0 = no PVS, 1 ≤ 10 PVS, 2 = 11–20 PVS, 3 = 21–40 PVS, and 4 ≥ 40 PVS) (Doubal et al., 2010; Charidimou et al., 2014). The presence of deep and periventricular white matter hyperintensity (WMH) lesions was visually evaluated using the 4-point Fazekas rating scale on axial FLAIR imaging (Fazekas et al., 1987).

### Statistical Analyses

All the data analyses were performed using SPSS software (version 25.0; IBM). The demographic, clinical characteristics and neuroimaging variables between the CAA, AD, and NC cohorts were summarized using the means and SDs for continuous variables, and counts and percentages were used for categorical variables. Bivariate comparisons were performed using the *t*-test and Mann–Whitney test.

The diagnostic sensitivity, specificity, positive predictive value (PPV), and negative predictive value (NPV) of the different methods used to differentiate CAA from AD were calculated with 95% confidence intervals (CIs). For the Boston criteria, only “probable CAA” was considered to be positive, and “possible CAA” or no hemorrhage was considered to be negative. For regional PIB uptake analysis, only occipital/global ratio was evaluated as the occipital predilection was most frequently reported in patients with CAA. For PET/MR, a positive diagnosis was made when a subject fulfilled both elevated occipital/global ratio and at least “possible CAA.” All tests of significance were two-tailed with a threshold for determining significance set at *p* < 0.05.

## Results

### Participant Demographics and Clinical Characteristics

**FIGURE 1:**
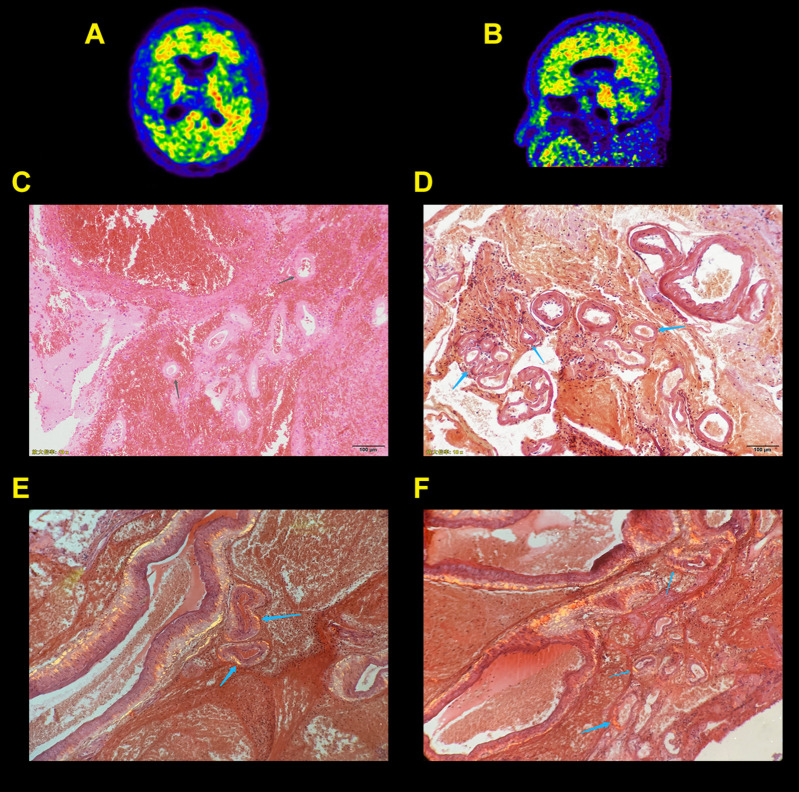
Probable cerebral amyloid angiopathy patient (CAA) with supportive pathology. Axial **(A)** and sagittal **(B)** Pittsburgh compound B (PIB) positron emission tomography (PET) scans show increased global neocortical PIB deposition with an occipital, temporal predominance. Photomicrographs show right temporal and parietal intracerebral hemorrhage in cerebral tissue sections stained with a hematoxylin and eosin stain **(C)**. A Congo red stain **(D–F)** demonstrates irregularly dilated blood vessels with fresh hemorrhage (gray arrows), and Congo red–positive blood vessel walls exhibit green birefringence with polarized light (blue arrows).

The demographics and clinical characteristics of CAA, AD, and NC subjects are summarized in Table 2. A total of nine patients with CAA, 15 patients with AD, and 15 NC subjects were included in this study. Mean ages ± ranges at the time of PET/MRI were 79.56 ± 8.90, 76.33 ± 11.06, and 78.00 ± 5.90 years of age, and the percentage of females were 22.2, 40, and 26.7% in the CAA, AD, and NC cohorts, respectively. The three cohorts differed significantly in most of the demographic and clinical characteristic data. NC subjects were more likely to have hypertension, diabetes, and dyslipidemia than CAA and AD patients. The blood vessel walls were Congo red-positive in one CAA patient (patient 6), indicating a pathologic diagnosis of CAA (Figure 1). Cortical superficial siderosis (cSS) was present in six CAA patients (66.7%); four patients had focal cSS, and two patients had disseminated cSS. Among the 15 AD patients, one patient (6.7%) had focal cSS and no history of ICH. NC subjects did not have cSS. All CAA patients had significant white matter hyperintensities (WMHs, Fazekas scale ≥ 2). AD patients had white matter changes that ranged from mild to moderate lesion loads. Mild white-matter ischemic changes were present in most of the NC subjects (Fazekas scale < 2).

**TABLE 2:** A comparison of demographic, clinical, and neuroimaging characteristics among the CAA, Alzheimer’s disease (AD), and cognitively normal healthy control (NC) cohorts.

Characteristic	CAA (*n* = 9)	AD (*n* = 15)	NC (*n* = 15)
Age, mean (range), years	79 (65–92)	76 (55–90)	78 (69–88)
Female, *n* (%)	2 (22.2)	6 (40)	4 (26.7)
Hypertension, *n* (%)	6 (66.7)	9 (60)	10 (66.7)
Diabetes, *n* (%)	3 (33.3)	3 (20)	9 (60)
Dyslipidaemia, *n* (%)	4 (44.4)	6 (40)	7 (46.7)
LVH, *n* (%)	1 (11.1)	0 (0)	0 (0)
Presence of lobar CMBs	9 (100)	8 (53.3)	4 (26.7)
≥5 CMBs presence, *n* (%)*	3 (33.3)	3 (20)	1 (6.7)
Presence of cSS	6 (66.7)	1 (6.7)	0 (0)
Focal cSS, *n* (%)	4 (44.4)	1 (6.7)	0 (0)
Disseminated cSS, *n* (%)	2 (22.2)	0 (0)	0 (0)
High grade	4 (44.4)	8 (53.3)	5 (33.3)
CSO-PVSs > 20, *n* (%)	2 (22.2)	2 (13.3)	1 (6.7)
BG-PVSs > 20, *n* (%)	2 (22.2)	6 (40)	4 (26.7)
Global PIB SUVR, mean (±SD)*	1.66 ± 0.06	1.86 ± 0.17	1.21 ± 0.06

*CAA, cerebral amyloid angiopathy; AD, Alzheimer’s disease; NC, cognitively normal healthy control; ICH, intracerebral hemorrhage; LVH, left ventricular hypertrophy; CMBs, cerebral microbleeds; cSS, cortical superficial siderosis; CSO-PVSs, centrum semi vale perivascular spaces; BG-PVS, basal ganglia perivascular spaces; WMH, white matter hyperintensity.*

Data are presented as the number (percentage) of patients unless otherwise indicated. *Significant.

### Lobar Cerebral Microbleed Distribution Patterns in the Cerebral Amyloid Angiopathy, Alzheimer’s Disease, and Healthy Controls Cohorts

**FIGURE 2:**
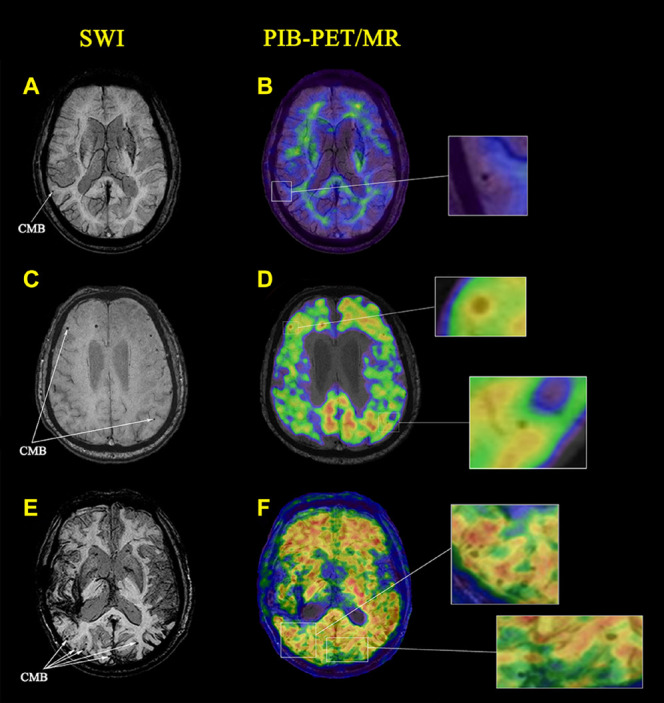
Cerebral microbleeds (CMBs) identified on images from magnetic resonance imaging (MRI) and PIB PET in a representative cognitively normal healthy control subject (NC) and patients with Alzheimer’s disease (AD) and CAA. **(A)** An axial susceptibility-weighted imaging (SWI) MRI scan from a 70-year-old woman with hypertension. A CMB is present in the right temporal lobe (white arrows). **(B)** A PIB-PET scan shows no lobar amyloid deposition (amyloid-negative) with only non-specific white matter deposition. **(C)** An axial SWI MRI scan from a 64-year-old man with long-standing hypertension. CMBs are present in the right frontal and left temporal lobes (white arrows). **(D)** A PIB-PET scan shows increased amyloid deposition in the right frontal lobe with no amyloid deposition in the left temporal lobe. **(E)** An axial SWI MRI scan from a 70-year-old woman without hypertension. CMBs are present in the bilateral occipital and temporal lobes (white arrows). **(F)** A PIB-PET scan shows widespread cortical amyloid deposition.

Representative SWI and PIB-PET images of NC, AD, and CAA patients with different CMB distribution patterns are shown in Figure 2. CMB distribution patterns differed significantly in the CAA, AD, and NC cohorts. CMB distributions were uneven between the left and right hemispheres with more CMBs in the right hemisphere than the left hemisphere. The number of lobar CMBs was greater in the CAA cohort. All CAA patients had lobar CMBs, and three CAA patients had more than five CMBs. CMB locations were concentrated to a greater extent in the occipital and temporal lobes relative to the frontal and parietal lobes. Among the 15 AD patients, eight (53.3%) had at least one lobar CMB, and three had more than five CMBs. Among the 15 NC subjects, four (26.7%) had lobar CMBs, and one had more than five CMBs.

### Pittsburgh Compound B Deposition at Cerebral Microbleed Sites

The local PIB-PET SUVRs at CMBs differed among the NC, AD, and CAA participants. As shown in Figures 2A,B, CMBs were present in the right temporal lobe. PIB was not concentrated at CMB sites in the NC subjects. Global PIB uptake in the NC subjects was low and could be easily distinguished from patients with AD and CAA. In the AD patients, CMBs were present in the right frontal and left temporal lobes. PIB-PET scans showed increased amyloid deposition at the right frontal lobe CMB sites, and no deposition at the left temporal lobe CMB sites (Figures 2C,D). The CAA patients showed widespread amyloid deposition in the cortical region (Figures 2E,F). The CMBs were present in bilateral occipital and temporal lobes and were associated with local SUVR concentrations.

We analyzed PIB-PET SUVR values at 150 CMB sites. PIB SUVRs were significantly higher at the CMB loci of the CAA patients compared with those of the AD and NC participants (1.72 ± 0.10 vs. 1.42 ± 0.16 and 1.17 ± 1.08; *p* < 0.0001).

### Global and Regional Pittsburgh Compound B Deposition

**FIGURE 3:**
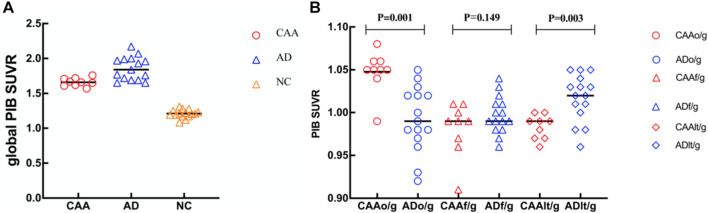
Dot plots of global and regional PIB standardized uptake value ratio (SUVR) comparisons in patients with CAA and AD, and cognitively NC subjects. **(A)** Global SUVRs were significantly higher in CAA patients compared with NC subjects and significantly lower compared with AD patients. **(B)** CAA patients had higher occipital PIB SUVRs (o/g) than AD patients and lower regional PIB SUVRs (t/g) than AD patients in the lateral temporal lobe. No significant differences were seen between the CAA and AD patients for the PIB SUVRs in the frontal lobe (f/g).

All CAA (*n* = 9) and AD patients (*n* = 15) were PIB-positive on quantitative assessments of the PET data. All NC (*n* = 15) subjects were PIB-negative, showing only non-specific depositions in white matter. Global SUVRs of participants with negative PET images ranged between 1.08 and 1.31, whereas those of a pooled group, including all patients with positive global PIB-PET, ranged from 1.58 to 2.17. Moreover, global cortical PIB depositions were significantly higher in CAA patients compared with NC subjects (1.66 ± 0.06 vs. 1.21 ± 0.06; *p* < 0.0001) and were significantly lower compared with AD patients (1.66 ± 0.06 vs. 1.86 ± 0.17; *p* < 0.0001; Figure 3A). These results were similar to those of a previous study (Johnson et al., 2007).

Although the PIB deposition was increased in both CAA and AD, the distribution patterns between these two groups were somewhat different. In CAA patients, there was a diffusive increase in PIB deposition across the other lobar regions. The lobar PIB deposition distributions were included in the occipital, frontal, lateral temporal, and parietal areas, with no significant differences between either hemisphere. However, PIB deposition was the highest in the frontal region and the lowest in the occipital region of AD patients. PIB deposition was found to have an occipital predilection in CAA patients (Johnson et al., 2007), which was confirmed by the ROI analysis of the present study. Relative to global cortical PIB depositions, occipital depositions were significantly higher in CAA patients than in AD patients (occipital/global ratio, 1.05 ± 0.02 vs. 0.99 ± 0.04; *p* < 0.001; Figure 3B). In contrast, the relative lateral temporal lobe deposition was significantly higher in the AD patients than in the CAA patients (lateral temporal/global ratio, 0.98 ± 0.01 vs. 1.02 ± 0.03; *p* = 0.003; Figure 3B). No significant differences in frontal depositions among the CAA patients and AD patients were found (frontal/global ratio, 0.98 ± 0.03 vs. 1.00 ± 0.02; *p* = 0.149; Figure 3B).

### The Diagnostic Value of Pittsburgh Compound B-Positron Emission Tomography/Magnetic Resonance for the Differentiation of Cerebral Amyloid Angiopathy From Alzheimer’s Disease

The sensitivity, specificity, PPV, and NPV of the Boston criteria, global and regional PIB SUVR analyses, and the proposed PET/MR-based method to differentiate CAA and AD patients are listed in Table 3. According to the ROC curve analysis, the optimal cutoff values for global SUVRs and occipital/global ratios were 1.765 and 1.035, respectively. Global PIB SUVRs showed good sensitivity and poor specificity (100 and 60%, respectively). Regional PIB analyses performed better than the “probable CAA” criterion of the Boston criteria (sensitivity, 88.9 vs. 77.8%; specificity, 86.7 vs. 66.7%). The combined method, using PET/MR, further increased the specificity, relative to the regional PIB analysis (sensitivity, 88.9%; specificity, 93.3%), as it ruled out one false positive case with no CMB.

**TABLE 3:** The sensitivity, specificity, and positive and negative predictive values of different diagnostic methods for differentiating CAA and AD.

	FN	TP	TN	FP	Sensitivity (%)	Specificity (%)	PPV (%)	NPV (%)
Boston only (“probable CAA”)	2	7	10	5	77.8 (40.2–96.1%)	66.7 (38.7–87.0%)	58.3 (28.6–83.5%)	83.3 (50.9–97.1%)
Global PIB only	0	9	9	6	100 (62.9–100%)	60 (32.9–82.5%)	60 (32.9–82.5%)	100 (62.9–100%)
Regional PIB only (occipital/global)	1	8	13	2	88.9 (50.7–99.4%)	86.7 (58.4–97.7)	80.0 (44.2–96.5)	92.9 (64.2–99.6%)
probable/possible CAA + regional (PET/MR)	1	8	14	1	88.9 (50.7–99.4%)	93.3 (66.0–99.7%)	88.9 (50.7–99.4%)	93.3 (66.0–99.7%)

*CMB, cerebral microbleed; PET, positron emission tomography; CAA, cerebral amyloid angiopathy; AD, Alzheimer’s disease; FN, false-negative; TP, true-positive; TN, true-negative; FP, false-positive; PPV, positive predictive value; NPV, negative predictive value.*

*Ranges in parentheses are 95% confidence intervals.*

## Discussion

The recent introduction of hybrid PET/MRI offers new opportunities for the diagnosis of age-related neural diseases. In this study, by combining Boston criteria with PIB-PET analyses based on PET/MR, we propose a diagnostic workflow to differentiate CAA from AD. To our knowledge, this is the first study combing these two modalities together for the differentiation of CAA patients and AD patients. ^11^C-PIB-PET/MR imaging shows an improved diagnostic accuracy for differentiating CAA from AD compared with separate MR and PET images. Although these results are derived from a limited number of CAA patients, they could still provide insights into the mechanism by which vascular amyloid leads to CMBs. They also inspire the design of multi-modality methods for diagnosing CAA.

Numerous prior manuscripts have assessed the diagnostic value of either amyloid PET or MRI in the differentiation of CAA against AD (Johnson et al., 2007; Lockhart et al., 2007; Ikonomovic et al., 2008; Dierksen et al., 2010; Ly et al., 2010; Yates et al., 2011; Baron et al., 2014; Farid et al., 2015; Graff-Radford et al., 2019; Sheikh-Bahaei et al., 2019), but it should be noted all of these studies investigated amyloid PET and MRI separately and none of them used integrated PET/MR systems to study CAA. The present study found that global amyloid deposition in CAA patients was higher than NC and lower than AD patients. Regionally, there was a difference between the pattern of deposition between CAA and AD patients with higher occipital deposition in CAA, suggesting that the relative predominance of occipital amyloid deposition may be related to the presence of CAA. Our findings confirm previous *in vivo* PIB PET reports (Johnson et al., 2007; Tsai et al., 2017; Sheikh-Bahaei et al., 2019); similarly, Tsai et al. (2017) found a higher global and regional amyloid deposition in patients with exclusively lobar CMBs than in exclusively deep CMBs patients. However, there was no significant difference in global and regional deposition between patients with CAA and age-matched HCs (Baron et al., 2014). The local amyloid deposition at lobar CMB sites was significantly higher in patients with CAA compared with that of AD patients. These findings also support the concept that amyloid mainly accumulates in vascular regions, while lobar CMBs occur preferentially in areas of concentrated amyloid.

Vascular amyloid is the pathologic substrate for CAA-related CMBs. PIB scans have been widely used to investigate the relationship between amyloid deposition and CMBs in patients with CAA. Prior studies showed that lobar CMBs were well correlated with amyloid burdens, and a topographic relationship was found to exist between regional PIB SUVRs and regional CMB densities in all the lobar regions (Yates et al., 2011; Graff-Radford et al., 2019). Tsai et al. (2017) investigated 250 patients with ICH using PIB PET and SWI scans and found that global amyloid deposition was significantly higher in patients with mixed CMBs distribution than that in patients with deep CMBs, but lower than in the lobar CMBs. In a longitudinal study of 11 patients with CAA, new CMBs occurred preferentially at sites of increased amyloid deposition (Gurol et al., 2012). However, these studies were based on regional analyses, providing circumstantial evidence regarding the association between CMBs and amyloid burden. In contrast, our study directly measured PIB deposition at CMB loci using colocalized PET/MR images with high precision. The results showed that the SUVRs were significantly higher at the CMB sites of CAA patients than AD patients, indicating different vascular pathologies between these two CMB types. As expected, amyloid deposition was very low at the CMB sites in the NC subjects, reflecting the pathology of hypertensive arteriopathy. CMBs were found in the bilateral occipital and temporal lobes of the CAA patients, and a remarkable concentration of amyloid deposits was observed at these CMB sites. In AD patients, the pathogenesis of CMBs was heterogeneous. There were locally high amyloid depositions at the CMB sties in the right frontal lobe, reflecting vascular amyloid-related CMBs. Conversely, amyloid was not concentrated at the CMB sites in the left temporal lobar region, indicating a hypertensive or other SVD pathology. These observations suggest PET/MR could help to distinguish underlying CMB pathologies, and benefit patients with possible CAAs as these patients are often easily mixed with AD patients with hypertension-related CMBs.

Using the Boston criteria, lobar intracranial hemorrhages (ICHs), which are large and symptomatic, and lobar CMBs, which tend to be asymptomatic hemorrhages, have equal CAA diagnostic value. However, ICHs typically do not occur in the absence of CMBs, and ICHs have a lower prevalence than CMBs. As a widely accepted and easily accessible neuroimaging marker in the clinical setting, the importance of studying the diagnostic value of lobar CMBs has been previously recognized (O’Donnell et al., 2000; van Rooden et al., 2009; Cordonnier and van der Flier, 2011). Recently, multiple studies have suggested that CMBs are restricted to lobar areas and more likely to be associated with CAAs (Knudsen et al., 2001; Johnson et al., 2007; Graff-Radford et al., 2019). Lobar CMBs have been shown to exhibit a predominantly posterior distribution across the brain lobes (Pettersen et al., 2008; Vernooij et al., 2008; Dierksen et al., 2010). According to a previous study, a diagnosis of probable CAA can be made based on the CMBs of hospital-based cohorts (Martinez-Ramirez et al., 2015). Similar to these studies, which reported CMB distributions, the present study reported that CMB distributions in CAA patients were higher in the occipital and temporal lobes than in the frontal and parietal lobes. However, the Boston criteria had a limitation in that the “possible CAA” category had a poor accuracy in diagnosing CAA compared with the “probable CAA” category (Knudsen et al., 2001; Linn et al., 2010). In this study, two patients in CAA group, eight patients in AD group, and four NC subjects all had one CMB. Some of these patients had hypertension, thus overlapping CAA and hypertension could not be excluded. The relationship between CAA and AD remains controversial. Incipient AD might also be present in patients suspected of CAA. The detection rate of CMBs is influenced by multiple factors, such as magnetic field strength, pulse sequence, and the populations studied. In addition, other pathologies, including microthrombi, calcifications, and air emboli, can also cause hypointensities on SWI, which mimics the signal voids due to CMBs (Schrag et al., 2010). All of these factors have led to the confusing CMB etiology and false positiveness, which limits the diagnostic utility of the Boston criteria.

Pittsburgh compound B-positron emission tomography is anticipated to increase the diagnostic accuracy of Boston criteria for the “possible CAA” category as it can provide the advantage of *in vivo* detection of amyloid deposition and directly bind to cerebrovascular amyloid deposits. According to a previous meta-analysis study, whole-cortical PET scans had moderate to good accuracy in differentiating CAA patients from NC subjects (Andreas et al., 2017). As reported in previous studies, the proportion of amyloid-positive elderly participants, despite normal cognitive condition, has ranged from 5 to 44.4% (Mintun et al., 2006; Johnson et al., 2007; Rowe et al., 2013; Baron et al., 2014). The prevalence of positive PIB observed in NC participants of this study is lower than that of previous studies. Our results showed that global-cortical PIB depositions were significantly higher in CAA patients than those in NC subjects (*p* < 0.0001). In the present study, all the NC PIB-negative participants were determined according to the reported SUVR cutoff of 1.5 (Jang et al., 2019), and may be related to a strict inclusion criterion of the participants in the NC group, and a negative PET could rule out all the subjects with CAA and mild AD. However, for PIB-positive participants, the locations of the global-cortical PIB depositions greatly overlapped in the CAA and AD cohorts. Thus, global amyloid-positiveness alone could lead to the false-positiveness caused by incipient AD pathologies, which limited the diagnostic utility of global PIB uptake.

In contrast, occipital/global ratios had a smaller overlap between CAA and AD patients relative to the frontal/global and temporal/global ratios, indicating that amyloid depositions generally favored the occipital lobe in CAA patients and relatively spared the occipital lobe in AD patients. Regional PIB deposition assessments could be promising for the differentiation of CAA from AD, given that previous reports suggest significantly higher occipital/global ratios in CAA patients than in AD patients (Johnson et al., 2007; Ly et al., 2010; Yates et al., 2011). The mechanism of the occipital predominance for amyloid depositions is not entirely clear; one explanation is that amyloid depositions reflect posterior circulation properties (Weller and Nicoll, 2005). A key issue is how to define PET cutoff values to distinguish CAA from AD patients when assessing regional PIB deposition. The cutoff value of the occipital/global ratio was based on the ROC curve, and a threshold value of 1.035 was obtained from nine CAA patients and 15 AD patients. Prospective studies in large samples and the use of advanced image processing methods may help clarify the pathological mechanism of occipital amyloid deposition and be used to differentiate CAA from AD. Moreover, using young HC group as reference to describe the prevalence of CMBs and assessing the association between CMB distribution and amyloid deposition on PET/MR will also be of considerable interest.

**FIGURE 4:**
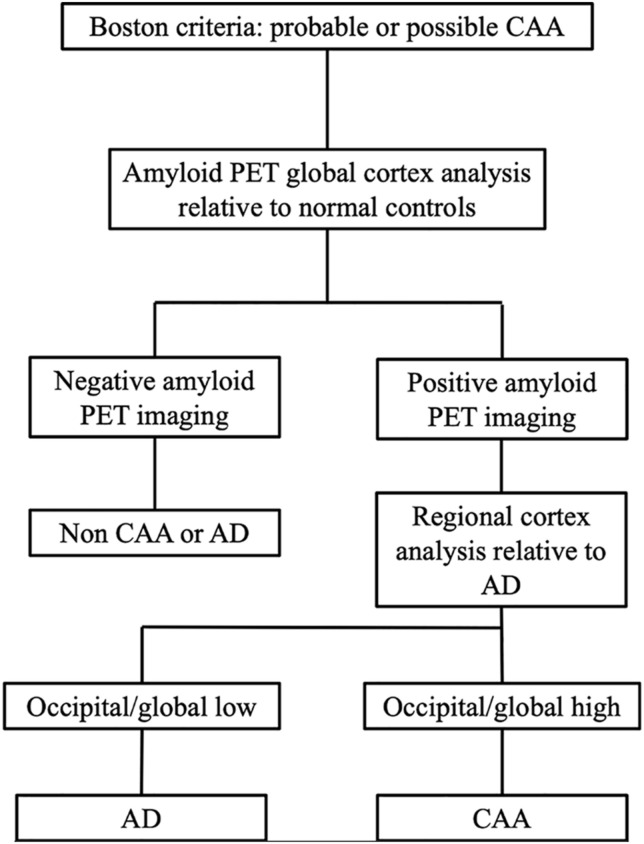
Proposed workflow using PIB-PET/MRI in patients to diagnose CAA vs. AD. This approach is based on three successive steps: (i) MRI images were used to detect suspected CAA patients; (ii) Global PIB uptake analyses were used to rule out patients without CAA or AD; and (iii) if global PIB positivity was found, then the regional PIB uptake patterns were assessed to differentiate CAA and AD.

Pittsburgh compound B is a non-specific imaging marker for both parenchymal and cerebrovascular amyloid. Therefore, negative amyloid PET rules out both CAA and AD. However, as discussed earlier, the overlap between CAA and AD limits the specificity of global PIB imaging in CAA as a clinical tool (sensitivity, 100%, and specificity, 60.0%). To overcome this limitation, we proposed a tentative approach to diagnose CAA with PET/MR, combining Boston criteria with PIB-PET analyses. In the proposed workflow of this study (Figure 4), when positive PET imaging was found, occipital PIB SUVR amyloid deposition was used to differentiate CAA from AD. Using this diagnostic workflow, one patient originally classified as possibly having CAA was confirmed to have CAA pathology due to high occipital/global tracer uptake. In contrast, four AD patients with more than two CMBs were ruled out due to the PIB distribution pattern of low occipital/global tracer uptake and high lateral temporal/global ratios. One “false positive” case was in a 75-year-old man with more than five CMBs and no history of hypertension. This patient had increased occipital/global tracer uptake in the occipital lobe, and the possibility of overlapping CAA could not be ruled out. The cause of false-positive CAA CMB site is PIB-PET imaging presumably reflecting the burden of neuritic plaques in occipital cortex and examining the spatial relationship between the vascular amyloid, but whether the relative predominance of occipital PIB deposition relates to the presence of asymptomatic CAA or co-occurs with AD pathology will require further investigation. When we combined the methods, the diagnostic accuracy was increased relative to the Boston criteria alone. The present pilot study enrolled a relatively small CAA sample, so confirmation of our observations is warranted. Although preliminary, this approach, based on simultaneously acquired PET/MR images, can be regarded as a step forward toward better differentiation of suspected CAA (either probable or possible) and AD according to a quantitative analysis, the use of an integrated PET/MR system is more convenient for patients in many aspects and potentially reduce the workloads and the cost of diagnosis. Further studies in larger cohorts are needed to validate our proposed diagnostic workflow for CAA.

Our study had a few limitations that should be noted. First, autopsies or histopathologic confirmations of amyloid plaque accumulation was not performed in all patients. CAA was defined primarily using modified Boston criteria and clinical symptoms. AD patients were diagnosed clinically before the PET study, which was conducted by certified physicians applying the NINCDS-ADRDA criteria. Using these criteria, the possibility of overlapping CAA could not be ruled out, thus weakening the results of this study. In addition, the patients with CAA and AD were relatively small in number and restricted primarily to a single site. These patients might not fully represent the whole CAA and AD population, which leads to possible selection bias. Therefore, further extensive, systematic, unbiased prospective studies are needed to assess the diagnostic value of the proposed diagnostic workflow for differentiating CAA and AD.

## Conclusion

We used PIB-PET/MR to detect cerebrovascular amyloid in patients with CAA, AD, and NC subjects, and our results indicate that the CMBs occur preferentially at loci with concentrated amyloid. By combining lobar CMBs with regional cortical amyloid deposition, the proposed workflow can further improve CAA diagnostic accuracy without the need for histopathologic confirmation. These findings improve our knowledge regarding the pathogenesis of CMBs and highlight the potential utility of PIB-PET/MR as a non-invasive tool to diagnose CAA in clinical settings.

## Data Availability Statement

Appropriate anonymized data can be made available to qualified researchers with a reasonable scientific proposal, by written request to the corresponding author. To gain access, data requestors will need to sign a data access agreement.

## Ethics Statement

The studies involving human participants were reviewed and approved by the Chinese PLA General Hospital Human Ethics Committee. The patients/participants provided their written informed consent to participate in this study. Written informed consent was obtained from the individual(s) for the publication of any potentially identifiable images or data included in this article.

## Author Contributions

YC, JL, and BX contributed to the conception and design of the study and wrote the first draft of the manuscript. ML revised the manuscript. LW, XL, and ZW contributed to the acquisition and analysis of the data. WJ and MZ provided the pathological picture and diagnosis. ML and BX provided critical revisions. All authors contributed to manuscript revision, read, and approved the submitted version.

## Conflict of Interest

ML is employed at Siemens Healthcare Ltd. The remaining authors declare that the research was conducted in the absence of any commercial or financial relationships that could be construed as a potential conflict of interest.

## Publisher’s Note

All claims expressed in this article are solely those of the authors and do not necessarily represent those of their affiliated organizations, or those of the publisher, the editors and the reviewers. Any product that may be evaluated in this article, or claim that may be made by its manufacturer, is not guaranteed or endorsed by the publisher.
